# Influenza vaccine uptake in Tunisia from two high-risk groups’ perception and attitudes: a qualitative study

**DOI:** 10.3389/fpubh.2023.1212431

**Published:** 2023-08-16

**Authors:** Aicha Boukthir, Hind Bouguerra, Afif Ben Salah, Astrid C. Erber, Sana Chaabene, Hayet Moussa, François Guillemette, Nissaf Ben Alaya, Jihene Bettaieb

**Affiliations:** ^1^Department of Medical Epidemiology, Pasteur Institute of Tunis, Tunis, Tunisia; ^2^Département de communication sociale, Laboratoire Interdisciplinaire de Recherche en Enseignement Supérieur (LIRES), Université du Québec à Trois-Rivières, Québec, QC, Canada; ^3^Observatoire National des Maladies Nouvelles et Emergentes, Tunis, Tunisia; ^4^Faculté de Médecine de Tunis, Université de Tunis El Manar, Tunis, Tunisia; ^5^Department of Family and Community Medicine, College of Medicine and Medical Sciences, Arabian Gulf University, Manama, Bahrain; ^6^Department of Epidemiology, Center for Public Health, Medical University of Vienna, Vienna, Austria; ^7^Centre for Tropical Medicine and Global Health, Nuffield Department of Medicine, University of Oxford, Oxford, United Kingdom; ^8^Département de Sociologie et d’Anthropologie, Institut Supérieur des Sciences Humaines de Tunis, Université de Tunis El Manar, Tunis, Tunisia; ^9^Département des Sciences de l’Education, Laboratoire Interdisciplinaire de Recherche en Enseignement Supérieur (LIRES), Université du Québec à Trois-Rivières, Québec, QC, Canada

**Keywords:** inflluenza, qualitative study, high-risk groups, vaccine uptake, barriers

## Abstract

**Background:**

Pregnant women (PW) and older adult with chronic diseases (ECD) are priority groups for the influenza vaccination. This study was designed to have a better insight into the influenza perceptions and barriers of the vaccine uptake from these groups’ perspectives.

**Methods:**

This qualitative study consisted of 20 focus group discussions (FGDs) enrolled from five governorates across the country (north, center, and south) between March 18 and July 10, 2019, in urban and rural areas. FGDs were conducted in Arabic (Tunisian dialect) and following the topic guide. Data were transcribed in the local language then translated into English and analyzed using Nvivo12 Software. This permitted the analysis thematic approach, using codes determined by the focus groups.

**Results:**

A total of 170 individuals participated in the FGDs (84 ECD and 86 PW). Both groups recognized the weakness of the immune system as key determinant for severity. While PW raised the lack of information about the vaccine, the ECD emphasized accessibility problems. Five main barriers to influenza vaccination were identified: cultural barriers and use of traditional medicine, misleading or lack of information about influenza and the vaccine, advice against its uptake, problems of availability and accessibility of the vaccine as well as mistrust towards the vaccine including adverse effects, vaccine composition and effectiveness.

**Conclusion:**

The study provided refined information from the perspectives of users to orient the policies regarding the promotion of influenza vaccine by decision makers among these two high risk groups.

## 1. Introduction

Influenza causes significant morbidity and mortality worldwide in addition to considerable economic costs (1, 2). Vaccination remains the most effective way to prevent illness or serious outcomes (3). In order of priority, WHO recommends annual vaccination for pregnant women (PW) at any stage of pregnancy, children from 6 months to 5 years, seniors (≥65 years), people with chronic conditions and health workers (4).

In Tunisia, seasonal influenza vaccine is provided free of charge in primary health care centers (PHC) part of the national Program of influenza prevention and control, targeted for older adult, people affected by chronic diseases, and health care workers (HCWs). Although identified as a high-risk group by the National Influenza Program, PW are not covered by the vaccination program. Vaccine is also available in the private sector and pharmacies (5, 6).

Little work has been done on influenza vaccine uptake. Like many countries of the Middle East and North African region, the estimated vaccination coverage rates remain low (7). Vaccine hesitancy is a barrier for high vaccination rates and is defined by the WHO Strategic Advisory Group of Experts (SAGE) working group as a “delay in acceptance or refusal of vaccines despite availability of vaccine services (8, 9).” This can be explained by various factors, especially personal beliefs on the safety and efficacy of the vaccine but also external factors such as the lack of awareness of the importance and accessibility of the vaccine (10–13).

Tunisia is currently working actively towards developing a seasonal influenza vaccine policy to make vaccine more accessible and to ensure optimal vaccine coverage. Decision makers should therefore better understand the knowledge and attitudes of target populations to develop the best methods of communication to reach these groups. Such strategic communications to educate the general public and vaccine target populations are critical to acceptance of vaccine (14).

In this context, the present study aimed to explore the perception of influenza vaccination and to gain a better understanding of the obstacles to influenza vaccine uptake for two specific target groups in Tunisia—older adult aged over than 65 years-old, especially those with chronic diseases (ECD), and pregnant women (PW). This study is part of a larger project, based on mixed research methods, of which quantitative study findings were recently published (15–17).

## 2. Methods

### 2.1. Study design and setting

This qualitative study was based on focus group discussions (FGD) among two target groups: pregnant women and older adult with chronic diseases. It was conducted in the three greater regions of Tunisia, which is categorized into five socio-economic regions, in each region we enrolled the most representative governorate that offers maximum variation needed to capture the perspectives of the study groups. A total of 20 FGD took place in five governorates across the country; governorates of Ariana and Siliana in the North, Sousse and Kairouan in the Centre and Gafsa in the South. In each of these governorates, 2 focus groups for each target population were performed, of which one in a rural and one in urban setting to ensure a better diversity of participants. Focus groups were conducted in primary health care centers in each governorate.

Investigation of the influenza vaccine uptake among another target group (healthcare workers) was performed in another quantitative study. We also conducted a qualitative study on this group using in-depth interviews, it will be published later. The selection of children from 6 months to 5 years is technically more challenging and ethically more demanding and requires a long process to obtain ethical approvals. This is why we focused on pregnant women and older adult who are reachable at the primary health care level. This work will open new research priorities targeting other high-risk groups or using different methodologies.

### 2.2. Sampling and participant recruitment

Purposive sampling was used to select focus group participants; 10 FGDs for each target group (20 FGD in total) were conducted. These groups were similar in composition, between 6 and 10 individuals to ensure equal voice to participants and to facilitate an equitable group dynamic. In the same way, similar distribution of EDC by sex was also ensured. To gain insight into the differences in perception and attitudes and enrich discussions, each FGD included both vaccinated and unvaccinated participants. The FGDs were conducted between March 18 and July 10, 2019. Permissions from regulatory authorities were taken prior to recruitment. Participants were invited to primary health care centers (HCCs) with the collaboration of health care professionals (HCPs), who were asked to identify participants on the day of their visit and regular checkup (antenatal care for pregnant women and chronic disease consultation for older adult with chronic diseases). Patients were informed about the study and invited to participate. Appointments were given for whom were interested.

### 2.3. Data collection

The FGDs were conducted in Arabic (Tunisian dialect) by two moderators helped by one note taker and following the topic guide using open questions. Based on this data collection technique, the researcher creates a conducive social environment in which group members are stimulated by the ideas and perceptions of their peers, (18). The staff involved was trained before and during the pilot phase to be familiar with the content and structure of the guides. The discussed themes of the guide were; (a) prevention: the means to protect oneself (disease in general and infectious diseases in particular); (b) knowledge about influenza: symptoms, modes of transmission, prevention, different forms of influenza, severity, sources of information; (c) exposure to influenza: perception of personal exposure; exposed populations and reason for this exposure; flu experience, treatment used; (d) the vaccine: (influenza vaccine knowledge; vaccine effect; evaluation of vaccine efficacy; where it is available; target population for vaccination); (e) vaccination; use of the vaccine; place where the vaccination took place; motivation to get vaccinated; reasons for vaccination hesitancy. (f) Awareness: effective means to inform and raise awareness about seasonal influenza vaccination.

All discussions were audio recorded and detailed notes taken before, during and after the FGD.

### 2.4. Data analysis

**Figure 1:**
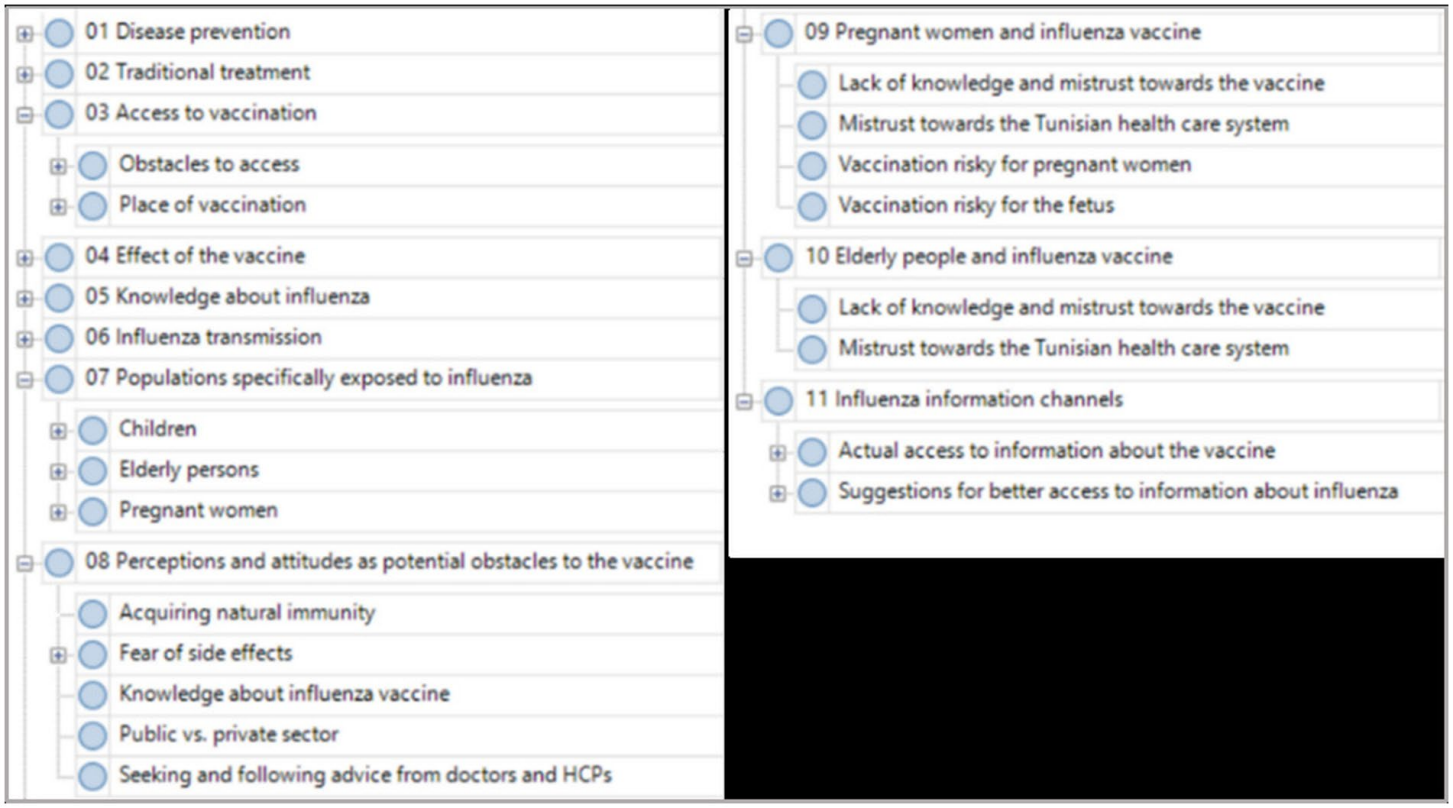
Coding framework.

Recorded data were transcribed in the local language then translated into English before analysis using QSR Nvivo Software. Transcripts were analyzed following a thematic analysis methodology for qualitative research to organize and explore the interrelations between the emergent themes. Iterative coding was used to identify major themes and concepts of the discussions as they emerge from the data. Codes were identified prior and during data collection (Figure 1).

Coding was conducted by two groups of researchers working independently and discussing regularly to resolve misunderstandings/misinterpretations and to modify and adapt the coding framework if necessary. Exploring the relationships between themes was also a part of the analysis.

**Figure 2:**
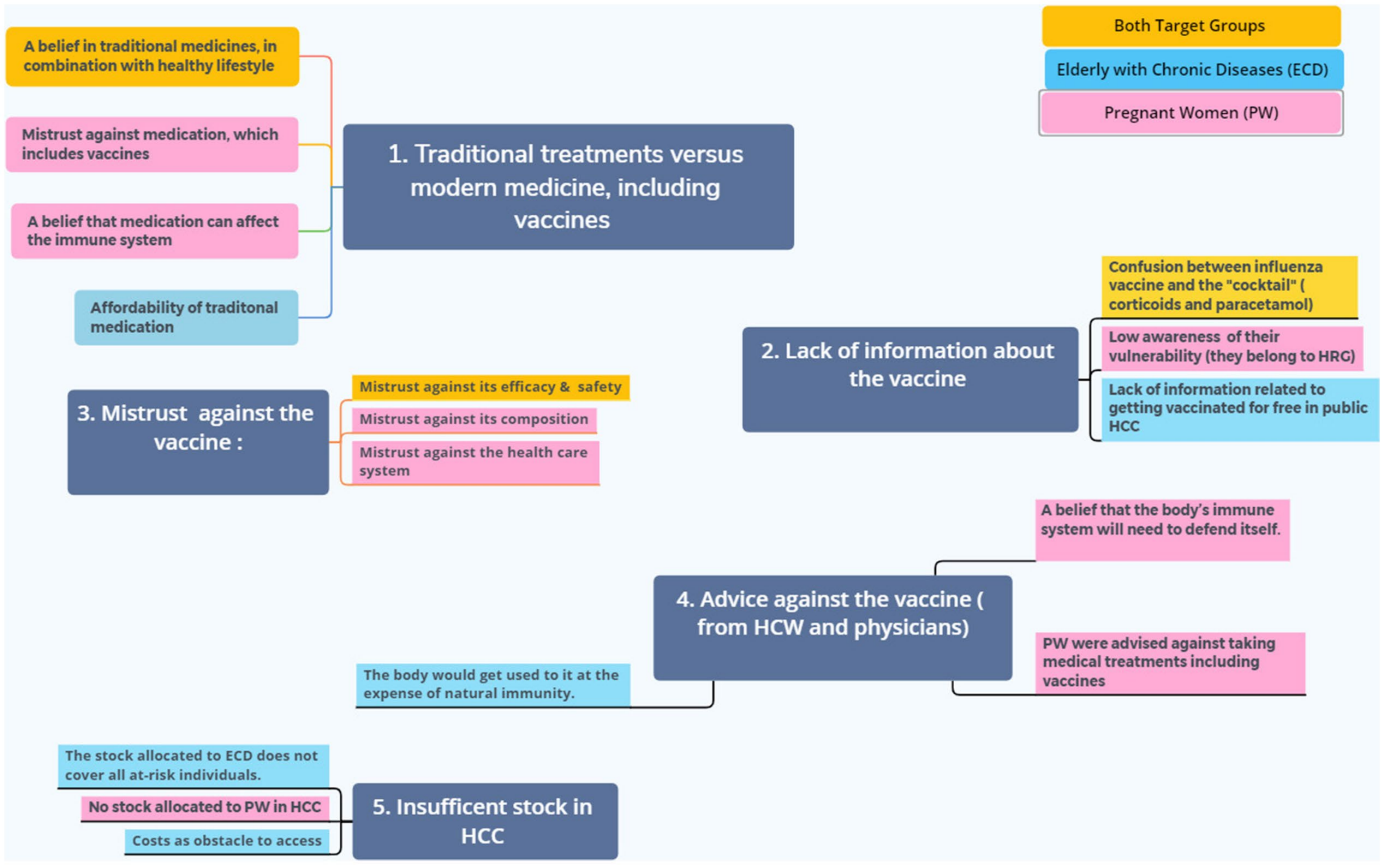
Main themes and sub-themes extracted from the analysis of transcripts of FGDs. Five main barriers to influenza vaccination were identified, subthemes in pink were expressed by pregnant women and those in light blue are related to ECD; some sub-themes are common for both high risk groups are colored in yellow..

### 2.5. Ethical considerations

An ethical approval (2018/40//I/LR161PT02) was obtained from the Ethics Committee of Pasteur Institute of Tunis. All participants were given an information sheet explained by investigators. To be part of the study, they had to sign the Informed Consent.

Interviews were conducted in closed rooms in the HCCs in order to guarantee their privacy. Only first names were used in the discussions and participants remained anonymous by replacing names by an ID during the transcription. Their identity was not disclosed for the analysis and reporting.

## 3. Results

### 3.1. Population characteristics

We conducted 20 FGD; 10 FGD among each target group, with a total sample of 170 participants; 84 EDC and 86 PW (Table 1). All study sites (Ariana, Gafsa, Kairouan, Siliana and Sousse) were covered (30 to 39 participants per governorate), as well as rural/urban areas.

**Table 1:** Frequency distribution of the target groups regarding their governorates and residence.

Governorate	EDC (*n* = 84)				PW (*n* = 86)		Total
	Men (*n* = 33)		Women (*n* = 51)				
	Rural	Urban	Rural	Urban	Rural	Urban	
Siliana	4	3	5	4	7	9	39
Ariana	4	2	5	6	4	9	30
Sousse	4	5	8	3	11	7	38
Kairouan	3	0	6	8	7	7	31
Gafsa	2	6	6	0	9	9	32
Total	17	16	30	21	45	41	170

EDC, older adult with chronic diseases; PW, pregnant women.

### 3.2. Perceptions of the disease and the vaccine

Information about influenza disease, are oriented by the biomedical knowledge, acquired either during the medical consultations or from media programs and social networks, especially for pregnant women. However, the disease is not well identified; although participants use in their discourse many medical nomenclatures, their information is partly based on lay interpretation and social representations shared collectively (culture, social groups, professional groups, etc.).

A major aspect was to explore participants’ experiences regarding the distinction between the common cold, and influenza. Participants have used the Tunisian Arabic term “brouda,” corresponding to the common cold, to refer to influenza. In line with this and in particular when assessing the severity of the disease, many participants spoke about a severe and a mild form in order to distinguish between the common cold and influenza. Influenza symptoms, sneezing, headache, joint pain, and coughing were described; fever is generally prominent when describing the severe form.

“*When it’s a cold, it disappears quickly, but if it is influenza, you will have fever, joint pain, so you have to go to the doctor and take medicine.*” (ECD Siliana)

“*We call it cold. It causes a cough, watery eyes and headache.*” (ECD Sousse)

“*Flu is the common cold, and the other forms are the influenza.*” (PW Siliana)

The severity of the disease is often related to the patient’s immune resistance, which is compromised particularly for EDC. For this group, their susceptibility was attributed to chronic diseases and a weakened immune system rather than age. Rarely, it was also attributed to the medication they have to take.

Pregnant women are also seen as particularly vulnerable to diseases in general, due to other conditions, changes to their bodies and to their immune system, and the additional burden of carrying a pregnancy and the baby delivery.

“*As PW, we are the most vulnerable to flu. During pregnancy, I cough so seriously, I can’t breathe normally, and I can’t control my bladder.*” (PW Gafsa)

“*Flu is dangerous, but it depends on the person. It depends on his immunity and his age also* … *Children can even die from flu, it can be very dangerous.*” (PW Kairouan)

### 3.3. Perception of influenza vaccine

We found that participants’ experiences and attitudes about Influenza vaccine among both high-risk populations are quite different. Most participants are aware of the vaccine and its efficacy, reporting that the vaccine works based on their own experience, but some of them pointed out that it might not have the same effectiveness every year.

“*I used to get the vaccine in October of each year before getting sick, it helps reducing the severity of the flu* […] *this year the vaccine was not effective enough, I had a severe flu*”. (ECD Sousse)

Some explained that the vaccine works, not by protecting them against infection, but by reducing influenza severity. Others perceive that influenza vaccine reduces the risk of being contaminated and of developing a complication but does not prevent the disease from occurring.

The ECD reported to be familiar with the influenza vaccine, with many of them having received a vaccination already. They often report being informed about it and its efficiency when attending HCC due to their chronic diseases. For them, HCPs Doctors are trustworthy sources of information.

“*Most of the time I get information from the TV, doctors and nurses show me how to take my medicines*”. (ECD Kairouan)

A few of them reported to refuse being vaccinated; reasons that were stated were rumors or personal opinions regarding potential side effects, or because the vaccine is not strongly recommended by HCPs.

### 3.4. Main barriers to influenza vaccination

The identified main themes extracted from the analysis of transcripts of FGDs represent the five main barriers to influenza vaccination, illustrated in Figure 2.

#### 3.4.1. Traditional treatments versus modern medicine, including vaccines

An important theme emerging in both groups was the belief in traditional medicines, often in combination with healthy lifestyle, and a general mistrust against medication, which includes vaccines. For pregnant women, the belief that medication can affect the immune system, reliance on natural remedies and healthy lifestyle and food are the main reasons. High cost is also mentioned.

“*Natural remedies are better than medicines, because medicines can weaken the immune system, we should make better food choices* […] *I use traditional remedies, especially hot and spicy soups.*” (PW Gafsa)

For certain respondents, poverty and the prohibitive cost of medications are the reason to choose traditional treatments. Others believed herbal treatments for symptoms’ relief are justified as influenza is a mild disease. Among the traditional treatments mentioned against flu symptoms were thyme, rosemary and eucalyptus, as well as cutting therapy whereby small cuts (scarification) were applied to the skin (mostly the forehead) to relieve symptoms such as headaches.

There were several opinions as to whether participants would use traditional treatment first or would seek HCPs’ advice. A shift in treatment seeking behavior, that is, to consulting doctors or going to hospital, was mostly noted by older adult participants.

#### 3.4.2. Misleading information/lack of information about influenza and the flu vaccine

There is a lack of information about the vaccine, which highlights the weaknesses of health authorities’ strategy to raise vaccine awareness, specifically for pregnant women who are not aware enough that they belong to high-risk groups who need to be vaccinated.

This was not the case of EDC who are relatively informed of their high risk, due to their regular contact with their doctor and HCW who provide information and ensure selection of patients to be vaccinated. However, some of them did not know that they can be vaccinated free of charge in the public HCC.

An interesting aspect emerged, is a frequent confusion between influenza vaccine and the “*cocktail*,” an injectable combination of corticosteroids and other components as paracetamol administered to treat influenza, bronchitis or other related symptoms, therefore mixing up prevention and symptomatic treatment.

This “cocktail” is sometimes prescribed by physicians but often given by nurses or in pharmacies; however, this “cocktail” is not recommended by the Ministry of Health, because it can delay appropriate case management particularly when the disease is severe and requires prescription of anti-viral drugs during a narrow window of time.

“*Many people are mistaken and make the cocktail of corticoids thinking it is the Influenza vaccine. They had a lot of problems afterwards.*” (PW Kairouan)

“*My husband takes the cocktail whenever he gets sick, and it works, I don't know if it was a* ‘*cocktail*’ *or an influenza vaccine.*” (PW Ariana)

#### 3.4.3. Advice against the vaccine

Pregnant women reported that they were often advised against taking flu vaccine. This advice was specifically from pharmacist, but even from health care professionals and media.

For many, this was a belief extended from the (meaningful) advice to not take medical treatments during pregnancy unless advised by a pharmacist or HCP.

“*I went to the pharmacy, but they said that I am pregnant and I cannot have vaccination. When they had started campaigns on TV, I went there with prejudices in mind.*” (PW Kairouan)

“*The doctor refused to give me the flu vaccine, since I’m pregnant. He said it may be harmful for my baby.*” (PW Siliana)

A surprising attitude found, in particular among the older adult, one respondent was advising his colleagues against the vaccine several times during the FGD.

“*Your body gets used to the vaccine and then you will need to get it every year.*” (ECD Sousse)

Advice against the vaccine was also due to a belief that the body’s immune system will need to defend itself or that the body would get used to it at the expense of natural immunity.

“*All it’s about is taking precautions* […] *The human body can defend itself. You will have the immunity and the strength to fight the disease.*” (ECD Siliana).

#### 3.4.4. Availability and accessibility of the vaccine (insufficient stock as obstacle to access)

Another main finding is about the availability and accessibility of the vaccine in health facilities where the stock allocated to ECD does not cover the entire at-risk individuals. An interesting context-specific aspect, related to reaching this target population; an invitation system makes it possible to manage the insufficient stock of doses provided by Ministry of Heath by prioritizing poly-morbid and oldest patients.

“*Last year, I got vaccinated against the flu here in the hospital. They said that every ECD should get the flu vaccine. But they didn’t bring it this year. So I’m very tired because of the flu this year.*” (ECD Kairouan)

For certain respondents, this lack will result in the purchase of an expensive vaccine at the pharmacy. Patients who seek medical care at public HCC often belong to low-income social classes. Therefore, participants noted that if the vaccine is not available for free, persons who are in need will not receive it.

“*I heard that we have to pay 14 dinars for it and I cannot afford this amount of money. If I get sick 4 times and even more, I will not take it.*” (ECD Ariana)

For PW, it was noted that the ministry of health does not allocate an annual influenza vaccine stock for them, which is attributed to the belief among health authorities that PW are known to be hesitant to the vaccine uptake and the potential allocated doses for them will not be used.

Many ideas raised around prioritization of health care versus universal affordability and coverage.

#### 3.4.5. Mistrust towards the vaccine (adverse effects, vaccine composition and effectiveness)

A rich variety of themes emerged in relation to mistrust towards the vaccine, mostly among PW and to a lesser extent among the older adult population. The first is the fear from related risk on their fetus. The inoculation of a product with an unknown clear composition for them, increase their hesitancy.

“*I fear for the unborn baby’s safety*” (Siliana PW)

Participants also noted several potential side effects and complications in relation to the vaccine. Perhaps the strongest one was a rumor that it could cause cancer. Others did not trust the vaccine composition or reported a fear of a lack of efficacy.

“*I comply with doctor’s advice. Some people believe that this vaccine causes cancer. But if my doctor recommends it, I’ll get vaccinated, but he didn’t.*” (ECD Ariana)

“*It was mentioned on TV. They said that it was a mix of medicines and that it was not recommended. You fear to take it because it may lead to other complications.*” (PW Ariana)

Another related theme is a mistrust against the vaccine as well as the health care system in general. One woman explained that she does not trust medicines and vaccinations, neither in the public nor in the private sector, as children are getting sick despite being vaccinated. Another PW discussed her fears related to the health care sector in general, and particularly in relation to vaccine safety.

The following interesting and complex comment by a pregnant woman explains the difference between vaccines available in the public and the private sectors. This might be due to vaccines procured from different companies, or just a subjective perception. The underlying issues seem complex and would merit further investigation.

Some older adult participants mention a good trust in “Vaxigrip,” provided by the public sector, in contrast to another, unspecified, type, provided by the private sector.

“*VAXIGRIP*” *provided by the Ministry of Health has no* [*side*] *effects and can protect us, more than once. I already have been taking that one, for two years now, but I won’t take the vaccine in private institutions.*” (ECD Gafsa)

### 3.5. Expectations of users/recommendations

Four main themes emerged during the FGDs as potentially mitigating the main barriers to influenza vaccination described above. Recommendations are presented as much as possible in participants’ own voices and summarized in Table 2.

**Table 2:** Summary of recommendations corresponding to identified themes.

	Theme		Recommendation
(1)	Lack of information about influenza	(a)	Inform the population about influenza, and the populations at risk
(2)	Lack of information about the vaccine		Inform the population about the vaccine and encourage its prescription
(3)	Advice against the vaccine		
(4)	Costs as obstacles to access	(b)	Make sure the vaccine is available and accessible
(5)	Insufficient stock as obstacle to access		
(6)	Traditional treatments versus modern medicine, including vaccines	(c)	Build more trust in the vaccine and the health care system
(7)	Mistrust towards the vaccine: a lack of efficacy, adverse effects and vaccine composition		
(8)	Mistrust against the health care system		

#### 3.5.1. Informing the population about influenza and the vaccine

When asked about the source of information about the vaccine, and optimal related communication channels, participants brought up several information channels and discussed their advantages and disadvantages focusing on their potential reach and trustworthiness. The main information channels mentioned were doctors and HCPs, Internet and social media, posters and brochures, TV and radio, rumors as well as awareness campaigns.

In general, doctors were seen as important sources of information, and especially trustworthy. Other HCPs such as nurses were mentioned as providing information. However, midwives were hardly reported, although they are seen as important points of contact for PW and could be a very valuable source of information. An additional aspect concerned the lack of time of HCPs to dedicate to each patient, mainly in the public sector, to inform patients about the flu vaccine.

“*Information is provided by the doctor and nurses. If the nurse doesn’t inform you, the doctor will.*” (ECD Gafsa)

TV was seen as a good mean of information, with a good coverage particularly among the illiterate population, but with a potential lack of trustworthiness. Radio is mentioned to a lesser extent, in particular in rural areas. On the other hand, Internet and social media were seen as convenient and accessible sources of information, as emphasized by PW, especially from the perspective of participants with high education. Posters and brochures in HCCs were appreciated in particular by older adult in urban areas. In rural areas, however, illiteracy poses a challenge for their understanding. Participants also reported being informed by relatives or acquaintances.

Awareness-raising campaigns were highly recommended, and very appreciated by our respondents. One older adult participant from a rural region remembered a health-related awareness campaign that took place on Saturday during the weekly market, which he considered a good means to reach the local population.

#### 3.5.2. Making the vaccine available and accessible

As discussed above, many participants mentioned the cost of the vaccine as a strong barrier to obtain and recommended adequate provision of the vaccine to ensure better accessibility.

Another recommendation given by participants is the timely information and availability of the vaccine, before the onset of the influenza season.

One interesting comment of a PW suggests a perceived contradiction between the universal indication of the vaccine, and its lack of availability and/or high costs, which raised her doubts about the credibility of the system.

#### 3.5.3. Building trust in the vaccine and the health care system

Participants reported a fear related to the vaccine composition, lack of efficacy and potential side effects. Specific information campaigns could help to address these issues.

A complex set of themes was brought up related to traditional treatments, and consequently, treatment seeking behavior, as discussed earlier. Aspects that could be clarified include traditional complements of medical treatments. A pregnant woman explained why she considers diagnosis by a HCP, as part of medical treatment, is important. This information is important to be considered in future communication plans.

“*I think that a campaign would be more effective, a campaign conducted in dispensaries, and mainly if it is at no cost … a sensitization campaign before the beginning of the season, people will be better informed*”. (PW Sousse)

## 4. Discussion

The present qualitative study is part of a research program, conceived with US-CDC, to provide a comprehensive situation analysis regarding the perspectives of the community in relation to influenza and related vaccine. It provides refined perceptions of end users about the barriers for an optimal uptake of the vaccine to guide preventive strategies against influenza and its complications particularly for vulnerable groups. Several quantitative studies are produced in this context (15–17). Triangulation of information from both approaches is a guarantee to get a thorough and deep understanding of the perceived bottle necks hampering the effective implementation of vaccination and other preventive pillars such as health education.

High vaccination rates benefit the individual vaccinated persons as well as those who are not vaccinated, e.g., due their age or poor response to vaccine, termed herd immunity (19, 20). For both high-risk groups, the communication related to the disease and the vaccine is still sub-optimal and requires further tuning to meet their needs. Better integration of this preventive tool in primary health care with more dedicated time during the management of patients is highly beneficial. Despite availability of the vaccine for EDC, timing and communication channels need to be more personalized.

However, we noticed differences in the degree of awareness about the influenza and related vaccine, where older adult seem to be less hesitant about the uptake of the vaccine and its benefits. They are more satisfied with the health providers and system than the pregnant women. This reflects the availability of a more structured preventive vaccination program for this high-risk group in Tunisia with availability of free vaccination in primary health care. For the latter, the vaccine provision is out of the patient’s pocket which reduces the uptake particularly in rural area and low socio-economic groups.

In line with results presented here, observed vaccine hesitancy is often depending on attitudes, but also strongly on local social, demographic and health-system-related issues (21–25). Studies regarding vaccine uptake have been conducted, e.g., in groups at risk (13), in health care professionals (HCPs) (26) or parents (27–29) and have shown that there is a need for context-specific as well as cross-disciplinary research which has been pointed out previously (25, 30, 31).

An improved understanding could then inform appropriate strategies for vaccine-related communication efforts and interventions, including communication strategies and messages (14, 25, 32–35). Often, those strategies are based on methods from marketing research (36, 37), such as a study by John and Cheney (35) who used audience segmentation based on attitudes, and potential promotions in order to increase influenza vaccination among individuals 65 years old.

Related to the information channels, some participants reported that they did not take the vaccine as they had received a recommendation against it, or it had not been specifically recommended to them by their HCPs. This finding was reported in a Moroccan study where some unvaccinated respondents complained that health providers had not explained anything about the vaccine or had advised them not to vaccinate (38).

This stresses the crucial role of health professionals as a driving force for vaccination; their recommendation was the main reason for vaccine acceptance among both PW and ECD in the quantitative studies part of this project (15, 16).

In order to overcome this, pharmacists and doctors, which are seen as trustworthy information channels, should be actively encouraged to recommend it, in particular to high-risk groups. For pregnant women, midwives have more opportunities to meet this group and could be the best to improve the knowledge about the risks and increase the uptake of the vaccine.

## 5. Strengths and limitations

To our knowledge, this is the first qualitative study exploring the perception and barriers to influenza vaccine uptake among two high-risk groups in the eastern Mediterranean region.

A prior study was conducted to assess acceptability of the monovalent A (H1N1) pdm09 vaccine among PW in Morocco (38). As many contextual factors are similar in the region, our results can be useful to provide insight into the perception and barriers of influenza vaccine uptake among these high-risk groups in other countries.

The large sample of participants selected from the northern, central and southern governorates of Tunisia provided a spectrum of opinions reflective of the different regions while asking comparable questions.

The main limitation was the lack of insights from high-risk groups of higher-income classes. In fact, having urban FGDs did not cover these groups since those who seek care from public HCC come almost from the same social and income class in both urban and rural areas. A complementary study by conducting FGD in high-risk groups at private HC facilities is therefore recommended.

## 6. Conclusion

This study highlighted the central themes to consider in future communication plans for older adult and pregnant women to clarify misconceptions around influenza and vaccination. It confirmed the role of health professionals as key information channels for the success or failure of this program.

The present study also stresses the high priority to ensure affordable influenza vaccine for pregnant women, to customize communication contents, timing and channels; to engage more effectively the health care professionals including midwives towards this strategy and to better adapt the organization of health services.

## Data availability statement

The raw data supporting the conclusions of this article will be made available by the authors, without undue reservation.

## Ethics statement

The studies involving human participants were reviewed and approved by Ethics Committee of Pasteur Institute of Tunis. The patients/participants provided their written informed consent to participate in this study.

## Author contributions

AB, HB, ABS, NA, and JB: conceptualization and methodology. AB, HB, SC, and HM: data curation and investigation. AB, HM, AE, and HB: formal analysis. ABS: funding acquisition. AB: project administration and writing—original draft. ABS, NA, and JB: supervision. ABS, FG, NA, and JB: validation. AB, HB, ABS, AE, FG, NA, and JB: writing—review and editing. All authors contributed to the article and approved the submitted version.

## Funding

This project was supported by the Task Force for Global Health under [grant number: F821E23E-52C0-4C51-8FC9-C356C34499B2]. The funding body had no involvement in the collection, analysis, and interpretation of data or in writing the manuscript. The primary granter of this research study is the DC National Center for Influenza and Respiratory Diseases.

## Conflict of interest

The authors declare that the research was conducted in the absence of any commercial or financial relationships that could be construed as a potential conflict of interest.

## Publisher’s note

All claims expressed in this article are solely those of the authors and do not necessarily represent those of their affiliated organizations, or those of the publisher, the editors and the reviewers. Any product that may be evaluated in this article, or claim that may be made by its manufacturer, is not guaranteed or endorsed by the publisher.

## References

[1] World Health Organization. Influenza. (2022). (Accessed December 25, 2022). Available at: https://www.who.int/teams/health-product-policy-and-standards/standards-and-specifications/vaccines-quality/influenza

[2] World Health Organization. Burden of disease. (2022). (Accessed December 25, 2022). Available at: https://www.who.int/teams/global-influenza-programme/surveillance-and-monitoring/burden-of-disease

[3] Centers for Disease Control and Prevention. Prevent seasonal flu. Centers for Disease Control and Prevention. (2022) (Accessed December 25, 2022). Available at: https://www.cdc.gov/flu/prevent/index.html

[4] World Health Organization. Influenza (seasonal). (2022) (Accessed December 25, 2022). Available at: https://www.who.int/health-topics/influenza-seasonal

[5] Ministère de la Santé. Guide de la surveillance de la grippe, 1ère Edition, avril 2016. (2022). (Accessed December 25, 2022). Available at: http://www.santetunisie.rns.tn/images/docs/anis/guidegripf6102016.pdf

[6] Ministère de la Santé. Point info Direction de Soins de Santé de Base, no1/2022. (2022). (Accessed December 25, 2022). Available at: http://www.santetunisie.rns.tn/images/pointinfosdssb01.pdf

[7] Al Awaidy S Althaqafi A Dbaibo G. A snapshot of influenza surveillance, vaccine recommendations, and vaccine access, drivers, and barriers in selected middle eastern and north African countries. Oman Med J. (2018) 33:283–90. doi: 10.5001/omj.2018.54, PMID: 30038727 PMC6047181

[8] Jarrett C Wilson R O’Leary M Eckersberger E Larson HJ. Strategies for addressing vaccine hesitancy—a systematic review. Vaccine. (2015) 33:4180–90. doi: 10.1016/j.vaccine.2015.04.040 25896377

[9] European Centre for Disease Prevention and Control. Vaccine hesitancy. (2022). (Accessed December 25, 2022). Available at: https://www.ecdc.europa.eu/en/immunisation-vaccines/vaccine-hesitancy

[10] Evans MR Prout H Prior L Tapper-Jones LM Butler CC. A qualitative study of lay beliefs about influenza immunisation in older people. Br J Gen Pract. (2007) 57:352–8. PMID: 17504584 PMC2047008

[11] Telford R Rogers A. What influences elderly peoples’ decisions about whether to accept the influenza vaccination? A qualitative study. Health Educ Res. (2003) 18:743–53. doi: 10.1093/her/cyf059, PMID: 14654506

[12] Kilich E Dada S Francis MR Tazare J Chico RM Paterson P. Factors that influence vaccination decision-making among pregnant women: a systematic review and meta-analysis. PLoS One. (2020) 15:e0234827. doi: 10.1371/journal.pone.0234827, PMID: 32645112 PMC7347125

[13] Bettinger JA Greyson D Money D. Attitudes and beliefs of pregnant women and new mothers regarding influenza vaccination in British Columbia. J Obstet Gynaecol Can. (2016) 38:1045–52. doi: 10.1016/j.jogc.2016.08.004, PMID: 27969559

[14] Mac Donald L Cairns G Angus K De AM. Promotional Communications for Influenza Vaccination: a systematic review. J Health Commun. (2013) 18:1523–49. doi: 10.1080/10810730.2013.840697, PMID: 24298886

[15] Kharroubi G Cherif I Bouabid L Gharbi A Boukthir A Ben Alaya N. Influenza vaccination knowledge, attitudes, and practices among Tunisian elderly with chronic diseases. BMC Geriatr. (2021) 21:700. doi: 10.1186/s12877-021-02667-z, PMID: 34911475 PMC8672335

[16] Dhaouadi S Kharroubi G Cherif A Cherif I Bouguerra H Bouabid L. Knowledge attitudes and practices toward seasonal influenza vaccine among pregnant women during the 2018/2019 influenza season in Tunisia. PLoS One. (2022) 17:e0265390. doi: 10.1371/journal.pone.0265390, PMID: 35316299 PMC8939791

[17] Cherif I Kharroubi G Bouabid L Gharbi A Boukthir A Ben Alaya N. Knowledge, attitudes and uptake related to influenza vaccine among healthcare workers during the 2018–2019 influenza season in Tunisia. BMC Public Health. (2021) 21:907. doi: 10.1186/s12889-021-10970-y, PMID: 33980192 PMC8116062

[18] Talbot N. Fortin, M-F. et Gagnon, J.(2016). Fondements et étapes du processus de recherche: Méthodes quantitatives et qualitatives (3e édition). Montréal, Québec: Chenelière éducation. Rev Sci Educ. (2016) 43:264–5. doi: 10.7202/1042088ar

[19] Omer SB Salmon DA Orenstein WA deHart MP Halsey N. Vaccine refusal, mandatory immunization, and the risks of vaccine-preventable diseases. N Engl J Med. (2009) 360:1981–8. doi: 10.1056/NEJMsa0806477, PMID: 19420367

[20] Kim TH Johnstone J Loeb M. Vaccine herd effect. Scand J Infect Dis. (2011) 43:683–9. doi: 10.3109/00365548.2011.582247 21604922 PMC3171704

[21] Omer SB Orenstein WA Koplan JP. Go big and go fast—vaccine refusal and disease eradication. N Engl J Med. (2013) 368:1374–6. doi: 10.1056/NEJMp1300765, PMID: 23574116

[22] Nessler K Krztoń-Królewiecka A Chmielowiec T Jarczewska D Windak A. Determinants of influenza vaccination coverage rates among primary care patients in Krakow, Poland and the surrounding region. Vaccine. (2014) 32:7122–7. doi: 10.1016/j.vaccine.2014.10.026, PMID: 25454875

[23] Ruiz JB Bell RA. Understanding vaccination resistance: vaccine search term selection bias and the valence of retrieved information. Vaccine. (2014) 32:5776–80. doi: 10.1016/j.vaccine.2014.08.042, PMID: 25176640

[24] Kroneman M van Essen GA John PW. Influenza vaccination coverage and reasons to refrain among high-risk persons in four European countries. Vaccine. (2006) 24:622–8. doi: 10.1016/j.vaccine.2005.08.040 16169638

[25] Larson HJ Jarrett C Eckersberger E Smith DMD Paterson P. Understanding vaccine hesitancy around vaccines and vaccination from a global perspective: a systematic review of published literature, 2007–2012. Vaccine. (2014) 32:2150–9. doi: 10.1016/j.vaccine.2014.01.081, PMID: 24598724

[26] Vasilevska M Ku J Fisman DN. Factors associated with healthcare worker acceptance of vaccination: a systematic review and meta-analysis. Infect Control Hosp Epidemiol. (2014) 35:699–708. doi: 10.1086/676427, PMID: 24799647

[27] Harmsen IA Mollema L Ruiter RA Paulussen TG de Melker HE Kok G. Why parents refuse childhood vaccination: a qualitative study using online focus groups. BMC Public Health. (2013) 13:1183. doi: 10.1186/1471-2458-13-1183, PMID: 24341406 PMC3878652

[28] Opel DJ Robinson JD Heritage J Korfiatis C Taylor JA Mangione-Smith R. Characterizing providers’ immunization communication practices during health supervision visits with vaccine-hesitant parents: a pilot study. Vaccine. (2012) 30:1269–75. doi: 10.1016/j.vaccine.2011.12.129, PMID: 22230593

[29] Zuzak TJ Zuzak-Siegrist I Rist L Staubli G Simoes-Wüst AP. Attitudes towards vaccination: users of complementary and alternative medicine versus non-users. Swiss Med Wkly. (2008) 138:713–8. doi: 10.4414/smw.2008.12423, PMID: 19043817

[30] Deml MJ Jafflin K Merten S Huber B Buhl A Frau E. Determinants of vaccine hesitancy in Switzerland: study protocol of a mixed-methods national research programme. BMJ Open. (2019) 9:e032218. doi: 10.1136/bmjopen-2019-032218, PMID: 31678955 PMC6830664

[31] Holt D Bouder F Elemuwa C Gaedicke G Khamesipour A Kisler B. The importance of the patient voice in vaccination and vaccine safety—are we listening? Clin Microbiol Infect. (2016) 22:S146–53. doi: 10.1016/j.cmi.2016.09.027, PMID: 27939015

[32] Nowak GJ Sheedy K Bursey K Smith TM Basket M. Promoting influenza vaccination: insights from a qualitative meta-analysis of 14 years of influenza-related communications research by U.S. Centers for Disease Control and Prevention (CDC). Vaccine. (2015) 33:2741–56. doi: 10.1016/j.vaccine.2015.04.064, PMID: 25936726 PMC5856146

[33] World Health Organization. WHO | research for universal health coverage: world health report 2013. (2013). (Accessed December 14, 2017). Available at: http://www.who.int/whr/2013/report/en/

[34] Centers for Disease Control and Prevention. CDC H1N1 flu|2009 H1N1 and seasonal influenza and Hispanic communities. (2010). (Accessed November 5, 2019). Available at: https://www.cdc.gov/h1n1flu/qa_hispanic.htm

[35] John R Cheney M. Resistance to influenza vaccination: psychographics, audience segments, and potential promotions to increase vaccination. Soc Mark Q. (2008) 14:67–90. doi: 10.1080/15245000802034721

[36] Gregg AP Klymowsky J. The implicit association test in market research: potentials and pitfalls. Psychol Mark. (2013) 30:588–601. doi: 10.1002/mar.20630

[37] Kotler PT Armstrong G. Principles of marketing. 17th ed Pearson (2017). 736 p.

[38] Lohiniva AL Barakat A Dueger E Restrepo S Aouad RE. A qualitative study of vaccine acceptability and decision making among pregnant women in Morocco during the a (H1N1) pdm09 pandemic. PLoS One. (2014) 9:e96244. doi: 10.1371/journal.pone.0096244, PMID: 25313555 PMC4196726

